# Preface to the Second Volume of the Medico-Chirurgical Review. (Analytical Series.)

**Published:** 1821-06-01

**Authors:** 


### PREFACE

TO THE

### SECOND VOLUME

OF

### THE MEDICO-CHIRURGICAL REVIEW.

### (Analytical Series.)

*ANOTHER year has rolled away—another volume has completed its orbit—and still the horizon of the Journal's circulation ceases not to extend. This, however, has been the* asserted *condition of all journals, at all periods—whether in their infancy, acmé, or decline. It will hardly be contended that we have not exhibited some authentic proofs, in our list of subscribers, that the prosperity of the work was regularly progressive.* Nor do we lay claim to any other merit than that of industry, common sense, and good intentions—ceding, without reserve, the palm of talent, genius, and erudition to each and every of our cotemporaries; satisfied as we are that, for the humble attributes abovementioned, there is ample scope for exertion, and perhaps some reward in store.*

*The object of this Journal, professed at first, and inflexibly pursued afterwards, is that of more equally balancing professional information among the different gradations of medical society, through the medium of an ANALYTICAL REVIEW which, rejecting all acerbity of criticism, directs its whole strength towards the selection and concentration of what may prove* useful *in the daily walks of practice. This task, simple and easy as it may seem, is one of no trifling labour and difficulty. Besides the necessity of some tact and natural sagacity in discriminating the grain amid the chaff, there is necessary, in addition, a clearness of conception, a facility of expression, and a command of language—powers that cannot always be assumed, at will, with the critic's chair or censor's rod.*

---

* *When the present Editor undertook the sole management of the Journal, on the 1st July,* 1818, *the sale was under* 200. *The quarterly return for No.* VI. *from Messrs. Burgess and Hill, on the 1st December,* 1821, *was* 1375 *numbers sold during the preceding quarter. For the truth of this statement the Editor pledges his word of honour, and refers any gentleman to the books of the publishers for its correctness.*

*There is another object of this Journal, not less important perhaps in its results than the diffusion of knowledge. It is that of gradually promoting harmony and liberality throughout the members of the profession, and suavity of expression among its public writers. On the list of those numerous causes which unfortunately operate but too powerfully in sowing the leaven of dissention in the medical world, must undoubtedly be placed that wanton severity of criticism, personal abuse, and effusion of private malevolence, which, at one time, disgraced the Journals and works of this country, and still continue to do so on both sides of the Atlantic. Every honorable mind must revolt against such conduct, and it is the duty as well as the interest of all ranks of the Profession to discourage whatever tends to loosen the bonds of medical society, lacerate private feelings, and give vent to the worst passions of the human heart.*

*In conclusion, we have to acknowledge that the patronage which this Journal has received is far beyond its real deserts—a proof that the Public has often taken the will for the deed, and rather held out a premium for future industry, than a reward for past services. Under the impression of this feeling, on our parts, it is not unreasonable to anticipate progressive improvement, especially when it is considered that no medical journal ever before had the same inducement to persevere, or yielded the same recompence for literary labour. We ran all the risk, and we reap all the profit—we commenced without assistance, and we continue without control—we had great labour in* establishing *the character of the Journal, we have deep interest in maintaining that character unblemished—our title page is unemblazoned with Ancestral Escutcheons, any honours we acquire therefore must spring from our own industry—the Journal acknowledges but one patron, the* Public*; it consequently has but one master to serve—one object to pursue. Under all these circumstances, our duty, inclination, and interest running in the same channel, the* Public *may be assured of undiminished exertion on our parts, and we shall calculate on the cheering influence of continued approbation from them.*

